# Infrastructure-Wide and Intent-Based Networking Dataset for 5G-and-beyond AI-Driven Autonomous Networks

**DOI:** 10.3390/s24030783

**Published:** 2024-01-25

**Authors:** Jimena Andrade-Hoz, Qi Wang, Jose M. Alcaraz-Calero

**Affiliations:** School of Computing, Engineering and Physical Sciences, University of the West of Scotland, Paisley PA1 2BE, UK; jimena.andrade-hoz@uws.ac.uk (J.A.-H.); jose.alcaraz-calero@uws.ac.uk (J.M.A.-C.)

**Keywords:** networking dataset, network control rules, network management, network optimisation, 5G, intent-based networking

## Abstract

In the era of Autonomous Networks (ANs), artificial intelligence (AI) plays a crucial role for their development in cellular networks, especially in 5G-and-beyond networks. The availability of high-quality networking datasets is one of the essential aspects for creating data-driven algorithms in network management and optimisation tasks. These datasets serve as the foundation for empowering AI algorithms to make informed decisions and optimise network resources efficiently. In this research work, we propose the IW-IB-5GNET networking dataset: an infrastructure-wide and intent-based dataset that is intended to be of use in research and development of network management and optimisation solutions in 5G-and-beyond networks. It is infrastructure wide due to the fact that the dataset includes information from all layers of the 5G network. It is also intent based as it is initiated based on predefined user intents. The proposed dataset has been generated in an emulated 5G network, with a wide deployment of network sensors for its creation. The IW-IB-5GNET dataset is promising to facilitate the development of autonomous and intelligent network management solutions that enhance network performance and optimisation.

## 1. Introduction

Network management and network optimisation are key aspects in the development of 5G-and-beyond (B5G) networks and their success and viability in supporting the forthcoming demands of communication services and applications. The AN revolution is poised to have a profound impact on B5G networks, ushering in a new era of smart connectivity and communication. As traditional networks are constrained by their limits in terms of system capacity, operation efficiency, etc., autonomous systems are emerging as a solution to address these challenges and unlock unprecedented levels of performance. This technology evolution leads to the introduction of new levels of intelligence and automation in the management and provisioning layers of the 5G network [1]. By leveraging advanced technologies such as AI, machine learning (ML) and software-defined networking (SDN), ANs can gain advanced abilities of self-management, self-optimisation and self-healing. A B5G AN refers to a network infrastructure that combines the capabilities of 5G-and-beyond technologies and autonomous networking principles. ANs are composed of virtualised components, automated agents and intelligent decision engines able to perform closed-loop controls [1].

In terms of B5G networks, an AN leverages the evolution of traditional cellular networks by incorporating advanced features such as network slicing, virtualisation, softwarisation and edge computing. In order for ANs to be established in our actual infrastructures, it is necessary to have management networks capable of carrying out all these advanced features. One of the paradigms commonly used nowadays is the Intent-Based Network (IBN). According to [2], an IBN is a network that can be managed using intent. The primary objective of an IBN is to establish an autonomic network by simplifying its management and operation. To this end, an IBN conceives the creation of a complete autonomous network framework [3], improving the robustness of the network and achieving dynamic operation and maintenance [4]. An IBN supports management functions guided by the use of intent. Intent is a high-level description of a set of operational goals and outcomes that a network should meet and is supposed to deliver, respectively [2]. It defines objectives and outcomes in a purely declarative manner, rather than specifying the detailed network configuration [5] or how to achieve it. Intents are then translated into network policies, which provide much more specific details concerning network configurations [3]. Such network policies will result in the execution of network actions, achieving the desired outcome specified in the intent. Examples of intents in a 5G network are as follows. (i) “Ensure the deployment of the network slice meets the specified service-level agreement (SLA) with regard to latency, bandwidth, and reliability”. (ii) “Assign the highest priority to critical application traffic, ensuring low-latency and guaranteed bandwidth, while maintaining a minimum level of service for standard traffic”. (iii) “Eliminate any traffic flow exhibiting malicious behaviour or unauthorised access attempts, without disrupting legitimate network traffic”.

ANs pursue Intent-Based interactions, moving from human–machine interaction to closed-loop resource interaction [1]. In this context, AI emerges to enable this transition. AI has become a key feature in both network management and optimisation for B5G networks. Meanwhile, before AI training and development, some data-related steps are indispensable. This includes not only the implementation and deployment of network sensors and data collectors but also the further processing and adequacy of the data. Such procedures allow not only data extraction coming from every resource of the network topology but also gaining significant insights on real-time networking processes [6]. This enhances network optimisation because such data can now be used in ML models to perform quicker and better decisions. Decisions that traditionally are taken by slow human interactions can now be autonomously performed by ML algorithms.

Despite all the benefits that ML-based solutions can bring to B5G networks, their practical implementation is difficult and introduces a number of challenges. First of all, AI systems need a wide variety of data for training. This implies having access to a realistic (real or emulated) network and also having the possibility and the necessary tools to access and extract real-time data. Another option is to utilise an existing reliable and adequate dataset. For the latter approach, the scarcity of public networking datasets is evident. Furthermore, most of them are out of date and unreliable. This is due to the speed of change that networks, especially cellular networks, experience over the years. This change in network behaviours and patterns demands more dynamically generated datasets [6]. Such new datasets reflect not only the traffic flows and different types of attacks but also the inventory of the network topology where the data are being captured. Thus, the datasets are infrastructure-wide aware, taking into account all infrastructure levels: network-level, node-level, interface-level and technology-level. The capture of such data will make these datasets comprehensive, reproducible, modifiable and extensible. The above challenges have motivated this research. This paper proposes and presents a novel comprehensive dataset for network management and optimisation purposes in AI-driven B5G networks. This dataset in composed of infrastructure-wide (IW) and intent-based (IB) data, extracted from a B5G network. Such data are extracted in real-time using a closed-loop framework.

The rest of the manuscript is organised as follows. Section 2 summarises the state-of-the-art related to existing available network-related datasets. In Section 3, the materials and methods used to create the proposed dataset are exposed. The study area, a 5G multi-tenant network, together with the data collection sources and network sensors, are presented. Section 4 describes the scenario emulation for the dataset generation. This section includes the implementation details and the design and execution of the experiments. Section 5 provides a detailed explanation of the dataset structure. The dataset analysis and validation is then presented in Section 6. The discussion is included in Section 7. Finally, the conclusion is provided in Section 8.

## 2. Related Work

In order to ensure that AI models are effective in addressing real-world B5G networking challenges, it is essential to have access to real and complete datasets. Such datasets must accurately represent the network traffic, network topology and activities that the model is intended to analyse and predict. In this section, a deep explanation of different networking datasets is presented. These datasets are described in Table 1, which contains different categories specified in rows. First, in row “Network type”, it is specified what kind of network infrastructure has been developed to generate the dataset. Then, some rows are designated to describe the dataset’s information. The “Topology” rows refer to the level of detail of the network topology reported in the dataset. We refer to the “infrastructure-wide dataset” as a dataset that includes information from all network layers specified in the topology rows. Finally, there are some rows dedicated to specifying whether the dataset has metadata and metrics according to the different infrastructure-level components: host, port (or network interface), data-plane technologies in each port, data flows, port queues and data-plane technology port rules. The datasets described are placed in the columns, divided into three different types. Type 1 includes datasets extracted from common network architectures such as LAN, military network or cloud. Type 2 focuses on IoT (Internet of Things) networks integrated into 5G networks and Type 3 collects datasets extracted from a 5G network. Below is a summary of them.

Starting with the datasets included in Type 1. The KDDCUP99 dataset was created in order to develop a network intrusion detector. This dataset has been widely used over the past years for anomaly detection problems [13]. More than 20 attack types are simulated in the dataset, which can be divided in four categories: Denial of Service (DoS), Remote to Local (R2L), User to Root (U2R) and probing. To achieve the generation of the dataset, a wide variety of instructions were simulated in a military network environment. However, this dataset has been detected to have significant issues that highly affect the performance of IDSs. To overcome such problems, other datasets were generated from this one. For example, the NSL-KDD dataset proposed by Tavallaee et al. in [14]. In [15], Ferriyan et al. proposed the dataset HIKARI-2021, an IDS dataset that presents encrypted network traffic in a real-world environment. The dataset is labelled with different network attacks. It has up to 86 features extracted with the Zeek tool, which includes hosts and flows metrics.

Focusing now on other type of network datasets, we have highlighted Type 2 in Table 1. In [9], Ferrag et al. present the Edge-IIoTset, a cybersecurity dataset of IoT and IIoT applications. The authors prepared a IoT/IIoT testbed with different IoT devices. In such a scenario, they identified and analysed different attacks related to IoT and IIoT connectivity protocols. Additionally, Koroniotis et al. present Bot-IoT [16], a network traffic dataset that includes Botnet scenarios in a realistic IoT network. It is an intrusion detection dataset that trains models to detect various botnet attacks in IoT networks. Both works come out with realistic and high-quality IoT network traffic datasets for NID. However, both of them are based only on flow metrics and are not infrastructure aware. Furthermore, another drawback is that neither of them have been deployed in a 5G network architecture.

Finally, Type 3 includes the 5G-NIDD dataset, presented by Samarakoon et al. in [17], a fully labelled dataset built on a functional 5G test network. The network has the presence of different attack scenarios and non-malicious traffic from real users. The dataset is focused on NID, with a total 112 features including flow metadata and metrics.

All these exposed datasets were created with the same purpose: attack and anomaly detection. For this reason, all of them have similar characteristics regarding the topology level and its metrics and metadata at both port and flow level. However, there is a need for a dataset that records the network topology in more depth, along with its intent-based data, that is, data that reflect metadata and metrics associated with network policies, which are linked with network intents. Thus, the dataset would provide not only information related to network monitoring, but also information related to network control. In addition, to the best of our knowledge, none of the datasets found include intent-based data in a 5G network architecture. A dataset with such specifications could be used for autonomous network management, control and optimisation. This has been the principal motivation of the current research.

## 3. Materials and Methods

In this section, the study area of the dataset is presented. This refers to a 5G multi-tenant and intent-based network. In Section 3.1, a brief explanation of the principal components of a common 5G network is introduced to better contextualise the problem to be addressed. This explanation provides the reader with a clearer understanding of the underlying sources of the various network features discussed in the following subsections. Section 3.2 provides an explanation of the proposed IBN. Finally, in Section 3.3, the data collection sources of the 5G-IB network are presented. This pertains to the extraction of essential data from various network sensors and the subsequent storage process.

### 3.1. Reference 5G Infrastructure Architecture

**Figure 1:**
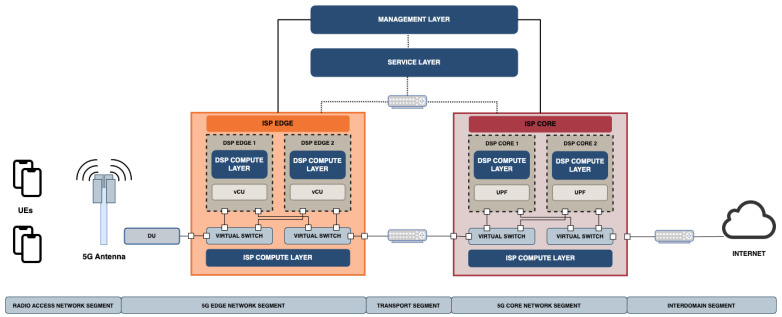
Overview of a 5G multi-tenant infrastructure data plane.

Figure 1 presents a reference 5G multi-tenant network infrastructure, where network services are softwarised and virtualised on the same physical infrastructure. In such infrastructure, the traffic remains isolated between tenants in consequence of virtualisation capabilities and traffic tunnelling across network segments. The 5G architecture is divided into five different network segments: Radio Access Network (RAN), Edge network segment, Transport segment, Core network segment and Interdomain segment. Only data-plane components have been represented in the figure.

The data flow traverses every segment, each serving a distinct purpose. Starting with the RAN segment, it represents the interface between user equipment devices (UEs) and the 5G network. It consists of antennas (Radio Units, RUs), the Distributed Units (DUs) and other equipment, responsible for transmitting and receiving wireless signals. The RAN is connected to the Edge segment through Centralised Units (CUs), which are virtualised and deployed in the Mobile/Multi-access Edge Computing (MEC) Network [18]. The Edge segment provides computing and storage resources closer to the end users, reducing latency and improving service quality. Edge and Core segments are connected through the Transport segment. The Core Network is the central part of the 5G network. It provides advanced functions such as session management, mobility management and authentication through different control plane components. It is connected to the Interdomain segment, which encompasses the interconnection between different administrative domains or service providers. Finally, as shown in Figure 1, the 5G system is composed of different stakeholders described in [5] by the 5G PPP (5G Public Private Partnership). These include Infrastructure Service Providers (ISPs) and Digital Service Providers (DSPs), both involved in the provisioning of network resources.

The presented architecture supports the quest for network automation that is achieved through cognitive loops. Autonomous network capabilities empower the network to self-manage and self-optimise its operations. Such automation is conducted by the deployment of different network layers, which are the compute, service and management layer. These network layers are represented as blue boxes in Figure 1. The compute layer plays a crucial role in enabling autonomous network capabilities as it provides the processing power and storage capacity necessary to support services and applications. The service layer encompasses the creation, deployment and management of services provided over the 5G network infrastructure. It focuses on delivering a large set of services that meet the diverse requirements of end-users and applications. This layer leverages automation, virtualisation and orchestration techniques to ensure efficient service provisioning, scaling and customisation. Finally, the management layer is responsible for supervising and controlling the network’s operation and resources. It makes use of advanced analytics and AI algorithms to monitor, analyse and optimise different network characteristics such as security, performance and resource allocation. This layer utilises data and information coming from the compute and service layers to create informed decisions and automate network management processes. The integration of these three layers in the 5G network allows the development of closed-loop capabilities such as adaptation to changing conditions, self-protection against attacks and optimisation of the resource utilisation [1].

### 3.2. Description of 5G Intent-Based Architecture

**Figure 2:**
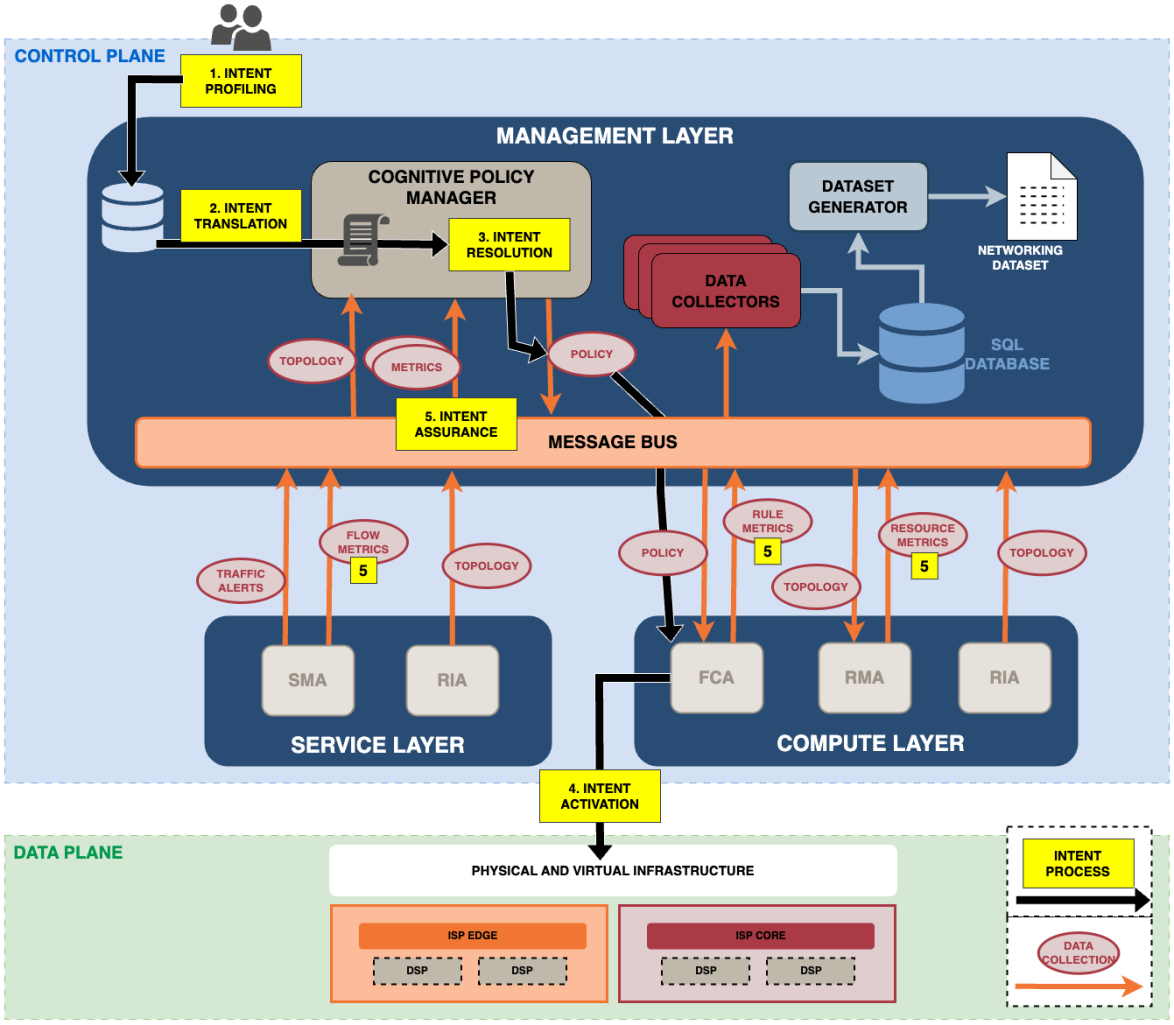
Design of the proposed IBN and overview of the different data sources of the IW-IB-5GNET dataset.

Figure 2 represents our IBN approach in order to achieve the closed-loop capabilities in the reference 5G network. Such an IBN approach automates the process of network configuration, provisioning and assurance by reducing human expert intervention. The proposed architecture consists of the three layers explained above, which are management, service and compute, in detail. Multiple network components (sensors and actuators) allocated in these layers work together to accomplish such autonomic features.

Our proposed IBN system establishes a closed-loop platform, where high-level service requirements are autonomously orchestrated and executed in the network. The full process since the intent is inserted into the network until the moment that is removed from it, consisting of five different steps [3]. Each of them are indicated in Figure 2 by yellow boxes and black arrows. The process is explained as follows:

1. Intent profiling. It is the first step of the IBN, where the user interacts with the system to specify the desired intent.
2. Intent translation. The intent statement is translated into a network policy, which will consist of a series of network rules and configurations. A policy is a set of rules defining what to do under what circumstances [19].
3. Intent resolution. It must be taken into account that multiple intents can happen in the network at the same time. For this reason, during the intent translation it is essential to prevent the network from leading to contradictory and conflicting network configurations.
4. Intent activation. The next step after confirming there are not any conflicts with other intent statements on the network is the intent orchestration and activation. This step includes the network configuration and provisioning of the requested network policy. As shown in Figure 2, this process results in the enforcement of a rule (or more than one) that will be reflected in the data plane.
5. Intent assurance. This ultimate step entails ensuring that the network indeed complies with the desired intent once it has been achieved. To accomplish it, our IBN comprises multiple sensors capable of monitoring the status of the network in near real time. Such components report metrics back to the management layer (see “5” yellow boxes in Figure 2), which is in charge of assuring the intent fulfilment. Depending on the type of intent, after Step 5, the intent-process will be finished or not.

With the completion of these five steps, the closed loop is complete. Such a loop operates as a self-contained system, responding autonomously to identified tasks and ensuring that the network aligns with the user-specified intent without requiring human intervention throughout the entire process.

### 3.3. Proposed Framework for Data Collection

This subsection describes the different software components necessary not only for the achievement of intent, but also for the dataset creation process. The data extraction process is detailed in Figure 2 by orange arrows and red circles. The infrastructure network approach is in line with the self-managed protection architecture proposed in [20]. It consists of a set of network software components distributed in the three layers previously defined working together to conform a cognitive closed-loop system. Each component runs a specific task and the combination of all of them leads to the accurate enforcement of network control rules. Such control rules have been previously translated from an intent. As depicted in orange in Figure 2, the communication and data exchange between components are facilitated by a message bus software through a publishing and subscription architecture. The red circles represent the type of data being exchanged in each case, while the orange arrows represent whether it is a subscription or a publication to that data exchange. The software components are described below.

- Resource Inventory Agent (RIA). It is a network component in charge of publishing topological network information in real time. Such information is related to both physical and virtual devices, ports and connections between ports and devices available on each machine. The RIA discovers the topology of the 5G network, where it is instantiated and publishes it for the rest of the network components. The performance and capabilities of this component are presented in [21].
- Security Monitoring Agent (SMA). It has two differentiated functionalities. First, it is in charge of enhancing and extending the capabilities provided by a traditional IDS. The reason behind this limitation can be attributed to the inadequacy of the capabilities possessed by traditional Network Intrusion Detection Systems (NIDS), which are unable to fully leverage the potential offered by the 5G infrastructure and the accompanying network information. Hence, the SMA works together with Snort [22] extending the information provided by this traditional NIDS. Second, it supplies information about network flows, providing an inventory of all flows traversing each of the network interfaces and traffic alerts. It also provides metrics associated with the reported network flows. This sensor and its capabilities are presented in [23].
- Resource Monitoring Agent (RMA). It allows the monitoring of different network resources. This agent extracts metrics from network devices, network ports (physical and virtual network interfaces) and data-plane technologies previously discovered and published by the RIA component. The monitored metrics are configured using a configuration file and can be easily extended by modifying such a configuration file.
- Cognitive Policy Manager (CPM). This component performs four differentiated tasks in order to generate network policies. First, it translates the user intent statements in network policies. At the same time, it is analysing flows and resource metrics provided by the SMA and RMA, together with the RIA’s spatial information. Thus, it is able to generate an intensive analysis of the current status of the network. Taking into account the network policy, it generates a decision using such analysis, which includes what action should be taken, where and with what data-plane technology. Such information will be used to complete the network policy. Depending on the intent type, the consequent policies can be diverse. Network policy examples are as follows: performing a drop, mirroring traffic, redirecting specified traffic, prioritising a concrete flow, etc. Once the action to be performed is decided, it performs the computation needed to complete the policy information, which is as follows: what action to enforce, in which interface of the network, how to enforce it and for how long will the policy be active. Once all this information is completed, it orchestrates the policy and publishes it in the message bus [20].
- Flow Control Agent (FCA). It exposes network traffic control capabilities to the management plane. The FCA is subscribed to the policy exchange and when it receives one, it translates the policy into specific network configurations and rules that can be executed by the network infrastructure in the data plane. The FCA is distributed across the whole infrastructure and it is an abstraction layer on top of different data-plane control technologies such as OpenFlow, SNMP, Linux Traffic Control (TC), Open Virtual Switch (OVS) and iptables. Once a rule is enforced in the network, the FCA also provides metrics associated with such a rule periodically. An extensive explanation of this agent and its performance can be found in [24].
- Data Collectors. All data exchanged on the network through the message bus, such as the topology, extracted by the RIA, the metrics, collected by the RMA, and SMA and the rule metrics reported by the FCA, are collected by the Data Collectors. The Data Collectors are in charge of transforming all data in SQL queries and insert them into a database in real time. As shown in Figure 2, there is a specialised collector for each data type. The collectors extract the information published by the network agents, adapt the data and store it into a SQL database. As a result, the management layer keeps an up-to-date database with all the network information published in real time.
- Dataset Generator. The Dataset Generator is the last component needed to have the resultant dataset and is the one in charge of creating the dataset. It is a software component that extracts, shapes, sorts and adapts to CSVs (comma-separated values) for the data stored in the SQL Database. The extracted data constitutes the different features of the dataset, which will be described in Section 5.

After providing an overview of the data extraction and collection framework, we will now proceed to delineate the specific requirements pertaining to the dataset. Additional details on the framework used can be found in our recent publication [25].

## 4. Scenario Emulation and Dataset Generation

This section describes the testbed infrastructure for the scenario emulation and details how the data are collected to generate the IW-IB-5GNET dataset.

### 4.1. Implementation Details

All software components described in Section 3.3 have been designed, deployed and validated in a realistic 5G mobile edge computing infrastructure. The vast majority are implemented in Java 17 (RIA, SMA, RMA, CPM and Collectors), with the exception of the FCA and the Dataset Generator, which are implemented in Python 3.8. The SMA component uses Snort 3.0 underneath to perform the attack detection. RIA utilises a collection of tools including OpenStack, OpenAirInterface 5G (W44 2022 or higher), LLDP, CDP and iproute2 (v1.9 or higher) in the Linux stack to detect the network topology. The message bus is implemented with RabbitMQ 3.6. The SQL database is MySQL 8.15. The Cognitive Rule Manager is a java implementation based on a MySQL 8 engine in order to allow the usage of SQL to reveal analytical, decision-making and planning policies. The FCA relies on Linux TC qdisk, OVS 2.17.3 and iproute2 (v1.9 or higher) to enforce the actions. The Dataset Generator is implemented in Python 3.8.10.

The emulation tool used for the creation of the network topology is the Common Open Research Emulator (CORE) [26]. It utilises Linux Network Namespaces (netns) to emulate (rather than to simulate) the different devices and networks that form the infrastructure. Each device or network operates within its own private network and process environments, while still sharing the same file system and kernel. Additionally, the Linux Ethernet bridging tools available in the Linux environment enable the emulation of any network type, including wireless mobile networks, thus realistically representing the detailed infrastructure described in this research. CORE was used for the implementation of a system that allows the creation, configuration, provisioning, emulation and execution of different experimental scenarios in 5G multi-tenant networks. More in-depth specifications of the system used can be found in [20].

In the context of our emulated 5G network, it is imperative to highlight the realism of the generated network traffic. The importance of this statement lies in the fact that the entire network is emulated, with the only exception of the link connecting the UE and the RAN component, which is simulated. Despite this limited simulation element, the veracity of the network traffic remains intact. This authenticity is maintained by meticulous prototyping of network components, protocols and behaviours, which ensures that the emulated traffic patterns closely reflect real-world scenarios. For instance, we use real core network elements provided by Osmocom (SGSNEmu [27] and GGSN [28]) and also by OpenAirInterface [29]. We also emulate multi-tenancy infrastructure making use of a custom Openstack Neutron-like SDN controller that populates isolated tenant networks using OpenVSwitch. Furthermore, the traffic is modelled to support both mobility and multi-tenancy through tunnelling protocols used in 5G architectures such as VXLAN and GTP. Full End-to-End topology emulation has been achieved using Linux containers to perform the deployment of each of the network functions on the relevant emulated devices to create a realistic 5G multi-tenant deployment. Therefore, all data traversing our emulated 5G network reflect genuine (non-synthetic) network traffic, and the emulated environment faithfully represents real-world network dynamics. This approach not only facilitates robust testing and analysis, but also reinforces the credibility of our emulation as a valuable tool for network evaluation and experimentation.

The experiments were run in a physical machine with an Ubuntu release 20.04 LTS distribution with kernel version 5.15.0. In terms of physical resources, it has a 56-core Intel(R) Xeon(R) CPU E5-2660 v4 @ 2.00 GHz and 128 GB DDR4 2400 MHz of RAM.

### 4.2. Experiment Design

Network security is one of the most important concerns of 5G operators [30]. For this reason, we decided to focus on security and collect the dataset based on the following intent: “Eliminate any traffic flow exhibiting malicious behaviour or unauthorised access attempts, without disrupting legitimate network traffic”. Distributed Denial of Service (DDoS) constitutes the 38.18% of the global network and application layer attack traffic according to Cloudflare radar [31]. In addition, the most popular DDoS attack type is UDP, being 54.4% of the total. In accordance with this fact, we decided to subject our network to UDP DDoS attacks, so that the resulting dataset records the state of the network while the intent is being fulfilled. Thus, the extracted dataset can be used to generate AI modules capable of making optimal decisions during the intent process. These decisions will be optimal as the specific network policy can be generated according to the state of the network at any given time rather than by default.

Multiple experiments have been designed and carried out in order to obtain a wide variety of data. The parameters studied in our research are as follows: the type of scenario executed, the type of policy to be performed within the network, the data-plane technology employed to execute the policy, the packet rate at which data are transmitted and the packet size used in these transmissions.

A total of four substantially different scenarios have been developed to achieve a more complete dataset. The four scenarios differ substantially in the number of edges, as well as the number of UEs connected to each edge. These are described below:

- Scenario 1. It is composed of two UEs and one edge. Thus, both UEs are connected to the same edge.
- Scenario 2. It consists of four UEs and two edges. There are two UEs connected to each edge.
- Scenario 3. It has eight UEs and two edges. Thus, there are four UEs connected to each edge.
- Scenario 4. It is composed of sixteen UEs and two edges. There are eight UEs connected to each edge.

**Figure 3:**
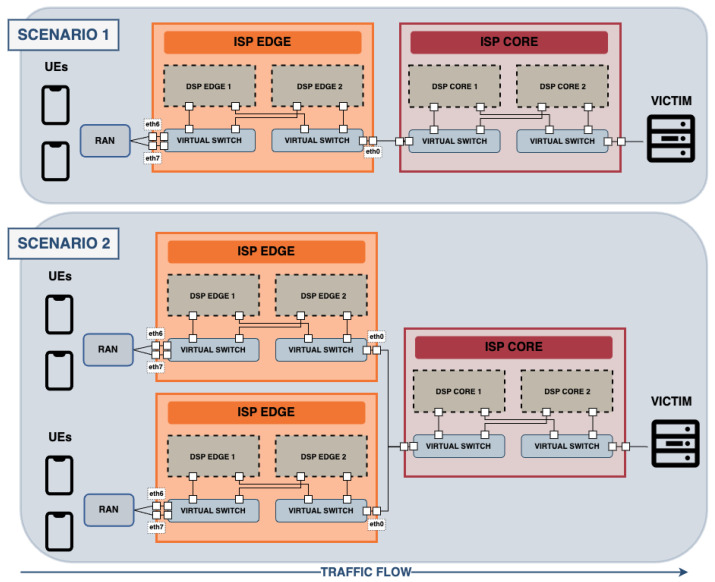
Architecture design of Scenarios 1 and 2.

The design of Scenarios 1 and 2 are displayed in Figure 3. Scenarios 3 and 4 follow the same design as Scenario 2, including a higher number of UEs connected to each edge. They have not been added in the figure for simplicity. For the same reason, network control layers have also not been included in the figure. Note that the number of UEs connected to the RAN has been chosen for specific use cases to have variety. However, this number can be extended, as well as the number of edges connected to the core network. This extension of the architecture is easily achievable using the CORE emulator, being a widely scalable network.

The type of action dictates the aspects of the network’s performance, behaviour and operations we will focus on and examine closely. In this present work, we have been focusing on the network’s behaviour while eliminating any traffic flow exhibiting malicious behaviour or unauthorised access attempts. This involves the choice of the policy to be carried out and the data-plane software technology with which to enforce it on the network. These decisions will profoundly influence the network behaviour. Three network software data-plane technologies have been studied in our research work, which are iptables, OVS and TC. Finally, variations in packet rate and size directly impact the performance metrics in terms of throughput, packet loss, bandwidth utilisation and congestion. For this reason, the variation of the packets traversing the network consisted of varying (i) the packet size: 32, 128, 256, 512 and 1024 bytes and (ii) the packet rate: 50 and 100 packets/s/UE. This provides a rate of packets per second reaching the victim between 100 in the lowest case and 1600 in the highest case (16 UEs and 100 packets/s), which are considered low-rate DDoS attacks, according to [32]. Specifically, constant low-rate DDoS attacks were generated. This type of attack consists of sending packets at a constant low rate [33].

The variation of these two parameters, together with the number of UEs, makes 40 executions for each data-plane technology, achieving a total of 120 executions for gathering data for the dataset. It is important to mention that depending on the number of UEs, each execution will contain different number of data files, according to the number of network rules active in the network. This means that the more UEs attacking the network, the more network policies from the intent will be processed to drop the malicious traffic.

### 4.3. Experiment Execution

This section explains how the data are generated and collected in order to create the dataset. As mentioned in the previous section, the final goal of each experiment is to gather data from different points of the network while malicious traffic is being eliminated from the network. The configuration of each experiment is the same. All the experiments have a duration of 3 min. Once all parameters specified in the previous section are set and the experiment is run, these are the steps followed by the autonomous system in order to compose the dataset:

1. Every software component involved in the cognitive closed-loop (described in Section 3.3) is started and operate in a standby mode.
2. The intent is inserted in the network using a command-line interface (CLI) (see Step 1 “Intent Profiling” in Figure 2). The intent is the same in every experiment: “Eliminate any traffic flow exhibiting malicious behaviour or unauthorised access attempts, without disrupting legitimate network traffic”.
3. The CPM translates the intent into a Policy template (see Step 2 “Intent Translation” in Figure 2), waiting for any traffic alert.
4. The UEs start sending two types of traffic. Bonesi [34] is the tool used for the DDoS attack traffic, while hping3 is used for the benign traffic (see Figure 3).
5. The software components in the service and compute layers of the ISP edge start extracting data as the traffic from the UEs cross the network. The exchanged data are shown in Figure 2 by red circles.
6. The SMA detects the malicious traffic and notifies the system of the attack through the message bus, using the traffic alert exchange.
7. The CPM performs all the steps described in Section 3.3 and generates a network policy. It is then published into the message bus. The policy specifies what to do, where and how. In our experiment, a drop action is performed in a network interface using concrete data-plane technology. The decision of the data-plane technology has been previously defined in the configuration of the experiment and is included in the policy template. On the other hand, the decision of the location (network interface) of where to perform the drop is calculated by the cognitive loop. Thus, depending on the data-plane technology to be used, the drop action will be performed on a particular network interface. In our specific scenario, as we are handling a DDoS attack, it is desirable to stop it as early as possible. This means that the drop action should be performed as close to the UEs as possible so that the malicious traffic does not traverse the network. Figure 3 highlights three different network interfaces where it is possible to perform a drop action. Data-plane technologies OVS and TC are available at interfaces eth0, eth6 and eth7. However, iptables will be available only at eth0. For this reason, when OVS or TC technology is selected, the drop will be performed at eth6 and eth7, respectively. On the other hand, in case iptables is selected, the drop action will be performed at eth0 as it is not feasible to enforce the action earlier in the network. After all these decisions and the policy is finished, Step 3 “Intent Resolution” is completed.
8. The policy is published in the message bus. An example of the policy message is shown in Listing 1.
9. The FCA receives the policy and translates it into a network rule, which is to drop the malicious traffic. As shown in Listing 1, the policy has also specified which network interface (eth6) and with what data-plane technology (TRAFFIC_CONTROL) to enforce the rule. As mentioned, the FCA is capable of performing the drop action with the following data-plane technologies: iptables, OVS or TC. This completes the Step 4 “Intent Activation” in Figure 2.
10. Once the rule is enforced on the network, the malicious traffic is dropped at the specified network interface by the data-plane technology defined in the policy. Meanwhile, all components continue reporting network behaviour metrics (“Intent Assurance”). With this final step, we can consider the loop has been successfully closed as now the network restores to its standby mode.
11. The experiment execution continues to complete in 3 min. During this time, the UEs continue sending malicious traffic. Such traffic is being stopped at the edge of the network. In the meantime, all the information and metrics generated during the execution of the experiment have been stored in the database and transformed into the resulting dataset.

**Listing 1:** Example of network policy.

{ “Policy”: { “actionType”: “INSERT”, “actionName”: “DROP”, “priority”: 1, “flowId”: “4BA92944”, “reportedTime”: 1659106981511 }, “Params”: [ { “paramName”: “interfaceName”, “paramValue”: “eth6” }, { “paramName”: “technology”, “paramValue”: “TRAFFIC_CONTROL” }, { “paramName”: “device”, “paramValue”: “edge1” } ] }

## 5. Dataset Description

This section provides an in-depth description of the dataset proposed and created in this research, with emphasis on its structure, the type of data gathered and the characteristics of its instances and features.

### 5.1. IW-IB-5GNET Dataset

The IW-IB-5GNET dataset is composed of several files. As it is an intent-based dataset, each file is associated with a network policy, translated from an intent. As a result, the number of files generated will correspond to the total number of active network policies in each experiment. For each file, the features are ordered topologically. This means that the features are organised in a top-down approach, starting with the device features, followed by the network interface features, data-plane technology ones, queues, network flows and network rules. The aggregation of the features corresponding to all levels of the 5G network topology makes it an infrastructure-wide dataset.

After the execution of each experiment described in Section 4, all the instances of each resulting file have been merged into a single csv file. This file forms the IW-IB-5GNET dataset. As a result of the 120 executions, a total of 700 csv files have been assembled. The dataset has a final dimension of 64,290 × 107, i.e., a total of 64,290 instances and 107 features. An excerpt of the dataset consisting of 1000 instances is available online (see Supplementary Materials at the end of the document).

### 5.2. Features Description

Dataset IW-IB-5GNET comprises 107 features related to the edge and core of a 5G network. Table 2 lists all the features and their positions in the dataset. The features are ordered topologically, starting with the metadata and metrics associated with the device (see 1–3 in grey in Table 2), followed by the device port (see 4–5, yellow), data-plane technologies and their queues (see 6–33, blue), traffic flows (see 34–49, red) and, finally, the network control rules (see 50–106, green and orange). Features 50 to 61 comprise the metrics and metadata of a particular network rule, enforced and monitored on a particular network interface (specified in feature 4, iface). Features 62 to 106 comprise analytical information related to all the network rules currently enforced in the network. With respect to RAN features, it is worth mentioning that we have decided to not include any value that is not coming from a trusted source. Thus, no physical layer feature is included, as it is the only link in the network that is simulated.

Based on their nature and the kind of information they represent, the features can be categorised into four different types as listed below.

- Boolean features. They refer to binary values, indicating the presence or absence of a particular attribute. The IW-IB-5GNET dataset has a total of four boolean features, enumerated in Table 3.
- Metadata features. They are composed of both categorical, numerical and text features. They are mixed together in the same category as they represent characteristics of every particular experiment. Most of them describe basic network characteristics and are essential to understand each specific use case. In addition, most of its values do not change throughout the experiments. However, it is important to keep them in the dataset in order to have a complete description of the network at each particular experiment execution. The metadata features are listed in Table 4. The table describes the data type of each feature. In the case of categorical features, the values they can acquire are specified. In addition, a brief description of what each of these features represent is included.
- Numerical features. They represent continuous or discrete numerical values. There are a total of 74 numerical features in the IW-IB-5GNET dataset. Such features are related to metrics measured in real time, at different network topology levels: device host, interfaces, flows, data-plane technologies, queues and rules. The names given to these metrics are sufficiently descriptive for the reader to know what they represent.
- Date/Time features. They represent specific points in time. There is only one date feature in the IW-IB-5GNET dataset, timestamp, which represents the moment when a concrete extraction has been performed. It is represented using the Unix timestamp.

To conclude the data description, as the reader can observe, there is no specific target associated with the dataset. This deliberate decision was made to ensure the dataset’s versatility and adaptability to various network control and management use cases. By not labelling the dataset with a specific target, it provides the freedom to utilise it for different network management and optimisation purposes, as described in Section 1. This approach allows us to explore and apply the dataset to address a range of specific needs, fostering innovation and flexibility in network management practices. Table 5 comprises a set of use cases for which our dataset could be used. It describes each use case, as well as its general purpose: management and optimisation. In addition, it is specified which label to give to the target column depending on the use case. Finally, the different features that, a priori, could be more relevant when analysing each specific use case have been emphasised. Note that this does not mean that these features are the only important ones, but rather that we try to reflect the versatility of the dataset in terms of its features when dealing with different use cases. Although the structure of the dataset applies to all these use cases, the current data obtained with the experiments described in Section 4 can be used for Security Management and QoS Optimisation. For the rest of the examples proposed in Table 5, more specific experiments would be necessary.

## 6. Results

In this section, we present the analysis and validation of the resultant dataset. This section presents the data obtained through tables and graphs, which can help better understand the dataset. The final objective of this section is to demonstrate the quality and reliability of the IW-IB-5GNET dataset. For the development of such analyses, we employed Python 3.9.6 and its libraries for data analysis: Pandas, Numpy, SciPy, Seaborn and Matplotlib.

### 6.1. Dataset Preprocessing

Prior to the IW-IB-5GNET dataset performance evaluation, we carried out some data preprocessing to ensure that the dataset was clean, consistent and suitable for analysis. This preparatory step was essential to mitigate the potential impact of noise, errors during the experiment executions and irregularities in the data, which can significantly affect the accuracy and reliability of subsequent analysis.

First, as mentioned in Section 5, it was necessary to consolidate all the CSV files from various experiments into a single file, simplifying the process of gathering statistics and conducting analysis. Once all data were in a single CSV file, the following were the four steps conducted in our data preprocessing pipeline:

1. Delete all rows whose columns were duplicates. This implies that all their columns have the same value.
2. Delete all rows that satisfied: activatedRuleTime = lastMatchedTime. Columns that satisfy this condition are the result of an invalid operation in the execution of the experiment, as they imply that a rule inserted in the network has not matched any packet.
3. Analyse and delete columns whose values were empty in all iterations.
4. Delete all rows whose columns contained negative values. None of the features were designed to be negative, so the presence of these values, if any, was due to an invalid operation during the experiment execution.

The preprocessing of the data resulted in the IW-IB-5GNET dataset, whose dimensions were already mentioned in the previous section: 64,290 × 107. Its memory usage is 51.2 MB. Analysis of the different types of features presented in Section 5 are discussed below in separate subsections.

### 6.2. Boolean Features Evaluation

Table 6 shows the statistical summary of the Boolean features in the dataset. It specifies the number of non-null values in each column, the number of unique values in the column, the most frequently occurring value in each column and the frequency of the top value. This summary is useful for gaining a quick understanding of the distribution and characteristics of boolean data within our dataset. For instance, it identifies the presence of the data-plane technologies OVS and TC in all the experiments executed where the drop action was performed (see OVS_activated and TC_activated in Table 6). On the other hand, it demonstrates that there are more cases where it is not possible to enforce a rule in iptables due to its absence on the monitored network interface (see IPTAB_activated). The statistics also reveal that most of the network flows dropped are in ingress (see sense).

### 6.3. Metadata Features Evaluation

**Figure 4:**
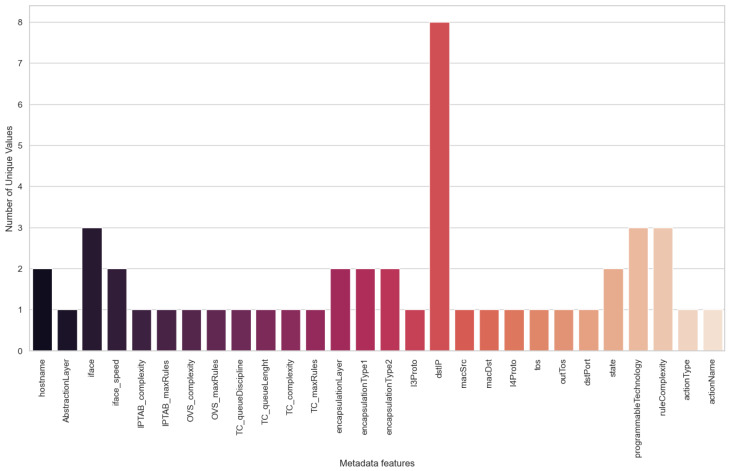
Number of unique values in each metadata feature.

As described in Table 4, 28 features comprise the variables categorised as metadata in our dataset. Most of them provide us with a better understanding of the network infrastructure, traffic flow characteristics and the network control rule active on each particular moment of the extraction. Figure 4 shows the number of unique values of each feature presented in Table 4. For instance, three different network interfaces were used throughout all experiments to drop the traffic flows. Other relevant features are the encapsulation of the traffic flows as they are presented. There were two different encapsulation types. The programmable technology give us the data-plane technology used to enforce the action on the network. Its value is three, as we have employed three different data-plane technologies: iptables, OVS and TC. Similarly, there were three different rule complexities, associated with the enforcement of each data-plane technology. Most of these features consistently maintain a uniform value throughout the entire dataset. This consistency stems from the fact that, as explained in the previous section, the metadata provides us with network-related information that remains relatively static over time. While this information might not hold immediate appeal, retaining it within the dataset is appropriate. This is because, if they were to conduct other types of experiments, this metadata would change, raising its relevance to the dataset.

**Figure 5:**
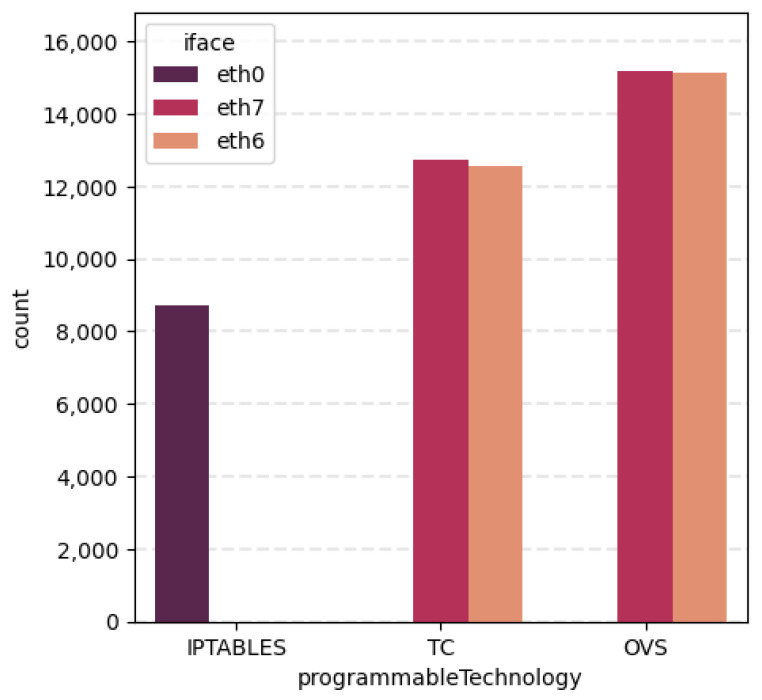
Association between interface and data-plane technology used in the action.

Focusing now on specific features, Figure 5 reveals interesting information connecting iface and programmableTechnology variables. The count plot is divided in the three data-plane technologies (iptables, OVS and TC) used for the enforcement of the network policy. In addition, such division is classified taking into account where this policy is performed in terms of its network interface. Thus, we can observe that all actions performed with iptables are enforced in network interface eth0, while TC and OVS vary between eth6 and eth7. This graph confirms the successful performance of the autonomous control loop, since the network policies are performed at the interfaces analysed in Section 4.3.

### 6.4. Numerical Features Evaluation

As described in Section 5.2, a total of 74 features comprise the numerical variables of the IW-IB-5GNET dataset. Some of them were removed during the data preprocessing process. Additionally, features 62 to 106 (see Table 2) already represent analytical information. Therefore, they were not considered in this data analysis.

**Figure 6:**
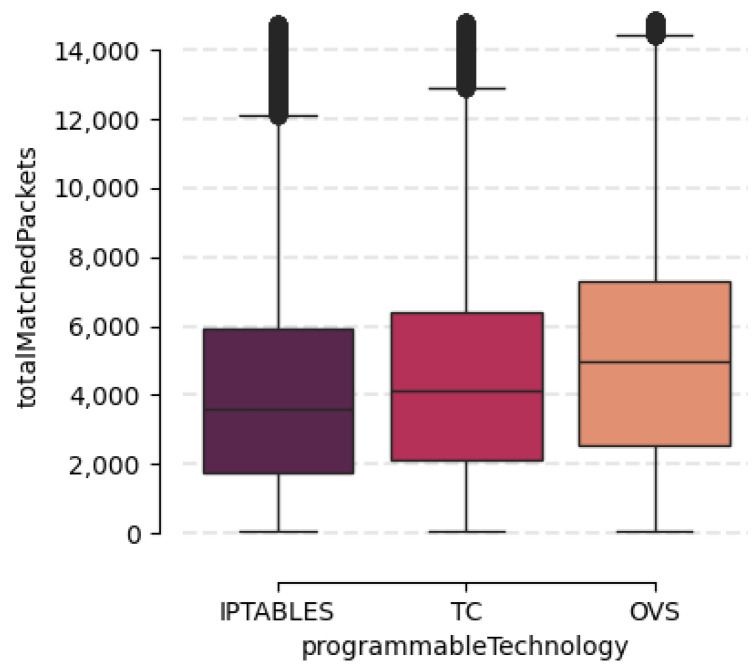
Box-plot of total matched packets depending on the data-plane technology used.

First, we start the data analysis with some descriptive statistics presented in Table 7. This table shows the statistics of the 22 non-null numerical features in our dataset. The first two columns indicate the feature name and its unit. The statistics include the mean, standard deviation, minimum and maximum measures. As can be seen in the table, most of these features are focused on monitoring packets or bytes per second traversing different points in the network. We can also observe information related to the number of packets traversing the network during the experiment and their size in bytes. With these measurements it is possible to identify congestion points along the network, as well as which data-plane technologies and on which network interface they are located. Finally, the last seven variables provide us with information about the effectiveness of the rule currently enforced on the network corresponding to the intent concerned. We have highlighted this effectiveness according to each of the data-plane technologies with which the drop action was carried out. This reflection can be seen in Figure 6. In this figure, a box-plot for each of the data-plane technologies is represented: iptables, TC and OVS. On the y-axis, on the other hand, the total number of packets that match the drop rule is represented. From this graph, it can be concluded that, for the same duration of the experiment (3 min), the number of packets that OVS is able to process is slightly higher than that of TC and, subsequently, that of iptables. These results indicate that OVS is faster in terms of the network rule processing time and can be taken into account when performing network optimisation tasks.

**Figure 7:**
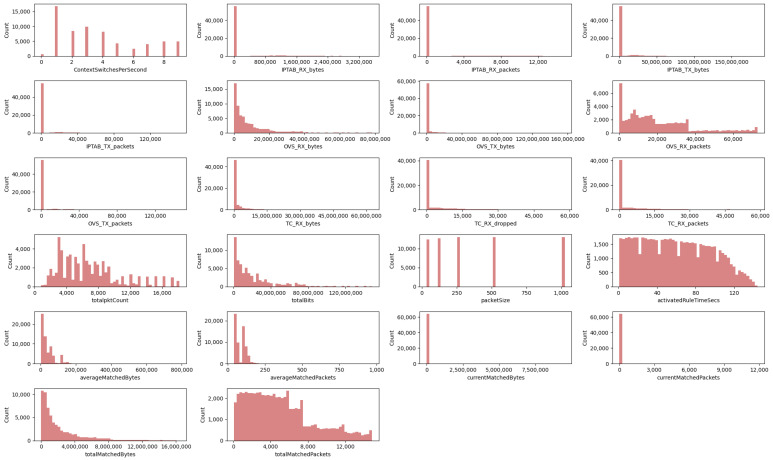
Histogram graphs of numerical features in the IW-IB-5GNET dataset.

Following the technical validation of our numerical features, we present Figure 7. It represents the data distribution graphs of the numerical columns in the IW-IB-5GNET dataset. A wide variety of shapes in the data distributions can be observed in the figure. First, we can identify discrete values such as ContextSwitchesPerSecond or packetSize. Focusing on the packetSize graph in Figure 7, the five different packet sizes chosen for the experiments can be observed. These are 32, 128, 256, 512 and 1024 bytes. Many histograms with exponential shapes are also present in the figure, such as the features totalBits and totalMatchedBytes. The totalBits feature indicates the number of bits actually traversing a particular network interface, while totalMatchedBytes designates the total number of bytes that match the network rule being monitored. Finally, we observe a variety of non-uniform shapes. Most of these have a high maximum value and much smaller cluttered values around it. Examples of these are totalpktCount or totalMatchedPackets. Looking at the graph of total matched packages in Figure 7, we can observe that the majority of values are around the mean, which is 5031. Additionally, its maximum value is 14,855 which indicates that in some of the experiments the rule matches a high amount of packets.

**Figure 8:**
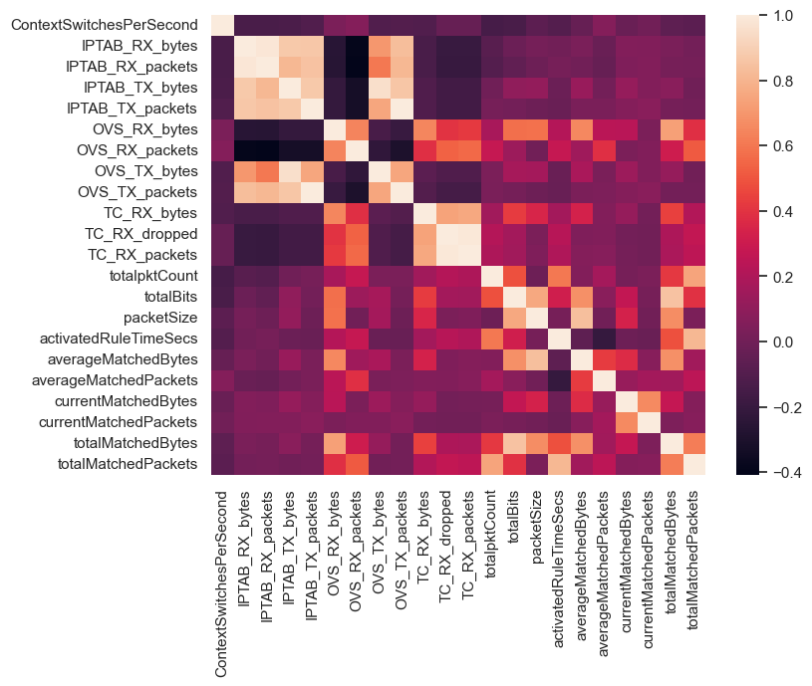
Correlation matrix composed of numerical features.

To conclude with the technical evaluation of the IW-IB-5GNET dataset, the correlation coefficients of the numerical features have been calculated. The technique used was the calculation of Pearson correlation coefficients (PCC), which measures linear correlation between two sets of data. The coefficient values vary between −1 and 1, which indicates the strength and direction of the linear relationship between two variables [35]. Figure 8 shows the correlation matrix obtained from these coefficients. It can be seen that features with high correlation are represented with a very light orange colour. In contrast, features with high negative correlation are represented with a dark purple. Features with low correlation between them are represented with intermediate colours. For instance, there is a clear positive correlation between bytes and packets of every pair of features associated with the same monitored instance (i.e., the more packets received, the more bytes received). An example of this can be found at the second and third features in Figure 8, IPTAB_RX_bytes and IPTAB_RX_bytes, respectively, where the very light orange colour representing their positive correlation can be noted. Focusing on very negative correlations, we look at the averageMatchedPackets feature with the activatedRuleTimeSecs feature. The more time a rule is active on the network, the fewer average packets matched that rule. In general terms, we observe a clear linear relationship in the lower right part of the correlation matrix. This corresponds to the metrics associated with the network rules, which are highly variable during the execution of the experiments. On the other hand, there is little linear dependence of the metrics associated with iptables with respect to the rest of the features. This is due to the fact that iptables is located at the egress interface of the edge network. Thus, traffic does not pass through in many of the experiments because the traffic is dropped before reaching the egress interface.

To summarise, the correlation matrix gives us a lot of information about the relationship of our data. Depending on the type of problem we expect to address using our dataset, we will need to pay attention to some patterns or others. For example, multicollinearity (two or more variables are highly correlated with each other) can be problematic in regression analysis, as it can lead to unstable coefficient estimates. Therefore, depending on the final objective of the dataset, the results will be taken as adequate or inadequate, and different actions may need to be taken.

## 7. Discussion

This section serves the purpose of consolidating and documenting the critical insights and deliberations derived from our research. This compilation is intended to provide a comprehensive resource for future researchers, enabling them to benefit from our considerations as a valuable foundation for their own scientific research.

Throughout the design and implementation of the proposed framework, we realised the great importance of performing mechanisms to align IDS from all components of the network topology. These are network flows, network ports, data-plane technologies available on each network port and device hosts. This is critical to allow the subsequent manipulation of features coming from each of these different entities. We have also realised and understood the real difficulty of achieving this intent-based, infrastructure-wide dataset, as it requires not only a complete infrastructure with a sufficient level of integration between the components, but also a complete and fully functional closed-control-loop running on top of it. Also taking into account that, in addition to the above, there is an automatic feature extraction system running.

In the context of our research, the utilisation of network emulators assumes a pivotal role in the generation of diverse scenarios for dataset collections. These emulators enable us to recreate controlled environments with precision and accuracy, facilitating the rigorous examination of various network conditions and their impact on our study. Finally, in our initial prototype, we recognised the significance of establishing interconnections between network components among timestamps, as they would play a crucial role in facilitating future AI training at a later stage. Consequently, a fundamental design principle that emerged was the incorporation of timestamps within every available interface in the proposed system. This design choice enables a traceability process. This approach aids in understanding both the context and timing of event generation within the system.

## 8. Conclusions

In this research, the need for a new dataset that could capture the complexities of B5G networks has been recognised, including their topologies at various levels and the dynamic nature of network control rules. This paper has presented a novel and comprehensive networking dataset, IW-IB-5GNET, which is infrastructure wide and intent based, addressing the pressing need for more robust and adaptable data-driven solutions in network management and optimisation in B5G networks. These solutions can be used by both ISPs and DSPs to improve the management and optimisation of their network policies in both the edge and core segments. Our dataset offers several key advantages in terms of network management and optimisation. Firstly, its infrastructure-wide reach ensures that it encompasses the entire network ecosystem, providing a broad view of network dynamics and status. This inclusiveness is vital as networks become increasingly complex and interconnected. Secondly, the dataset is intent based, achieving the fact that it not only documents the technical aspects of the network but also takes into account the underlying intents and control actions that drive network configurations and policies. It is important to highlight the nature of this dataset, which has been extracted from a closed loop in a B5G network.

The empirical and analytical results show the wide variation in the data distributions, as well as their most common values and linear correlations. These results show the state of the network at its different layers, from which valuable performance metrics are extracted. In particular, Table 7 and Figure 6 and Figure 7 allow it to perform an exploratory analysis of the data, an indispensable step prior to the implementation of any model. In addition, Figure 8 will help us to perform actions such as dimensionality reduction to optimise data-driven models. The results can be very useful for the generation of AI-based models to optimise network policies, as well as AI models to improve QoS, for example, the creation of a classification model to optimise the rules currently enforced in the network, so that the model can predict which technology is the optimal one to perform a network policy. Another example would be a model capable of detecting network rules that are not being used and, therefore, can be removed to improve network policy congestion. Nevertheless, our dataset also has some limitations. We are aware that it has limited scale, not being wide enough to capture yet the whole complexity of real-world scenarios. Furthermore, it lacks diversity in terms of intent coverage, focusing on a particular type of intent for now.

In future work, we will further explore the potential of the IW-IB-5GNET dataset. We will explore its applicability in a variety of domains, with a particular focus on the challenges of network management, optimisation and QoS. We will not only evaluate its effectiveness, but also apply well-known AI models. In addition, it is our aim to work on the limitations highlighted above, extending the scale and the coverage of the dataset. Not only by increasing the type of DDoS attacks, but also by applying other types of intent statements.

## Figures and Tables

**Table 1:** Table comparing different networking datasets (np: not provided) (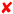: No, 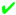: Yes).

		TYPE 1		TYPE 2		TYPE 3	
		KDDCUP99	HIKARI-2021	Edge-IIoTset	Bot-IoT	5G-NIDD	Ours
Network Type		Military	LAN	Edge-IoT	IoT	5G	5G
Dataset info	Extraction tools	tcpdump	tcpdump, Zeek	Zeek	pcap capturing	pcap capturing	Linux scripts, Python tools
	Purpose	NID	NID	NID	NID	NID	NMC *
	Format	csv	csv	csv	different sets	pcap, csv	csv
	Resolution	n.p	n.p	timestamp	timestamp	timestamp	timestamp
	Simulated	Yes	Partial	No	No	No	No
	Features	42	86	61	n.p	112	101
	Year	1999	2021	2022	2019	2022	2023
	Available	[7]	[8]	[9]	[10]	[11]	[12]
Topology	Host	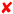	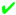	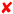	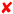	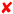	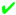
	Port	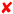	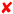	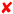	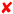	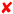	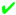
	Technology	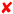	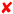	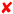	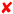	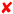	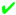
	Flow	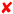	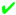	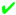	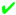	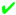	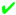
Metadata	Host	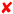	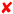	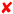	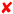	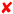	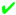
	Port	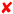	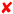	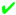	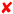	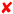	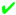
	Technology	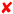	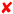	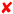	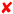	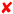	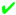
	Queue	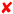	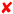	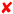	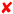	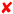	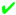
	Flow	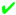	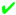	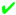	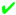	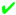	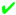
Metrics	Host	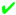	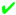	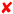	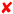	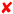	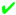
	Port	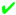	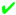	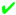	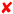	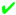	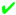
	Technology	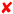	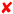	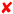	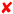	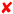	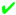
	Queue	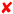	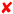	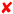	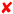	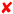	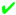
	Flow	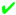	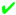	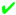	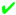	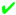	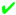
	Rule	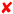	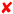	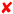	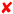	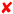	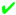

* Network management and control.

**Table 2:** List of features in IW-IB-5GNET dataset. Color indicates topology level: (grey-device), (yellow-interface), (blue-technology), (red-flow) and (green, orange-policy).

No.	Feature	No.	Feature	No.	Feature	No.	Feature
1	Hostname	28	TC_rx_bytes	55	currentMatchedPackets	82	OVS_mean_crr
2	ContextSwitchesPerSecond	29	TC_RX_dropped	56	lastMatchedTime	83	OVS_median_crr
3	AbstractionLayer	30	TC_RX_packets	57	ruleComplexity	84	OVS_st_crr
4	Iface	31	TC_TX_bytes	58	totalMatchedBytes	85	OVS_q1_crr
5	Iface_speed	32	TC_TX_dropped	59	totalMatchedPkts	86	OVS_q3_crr
6	IPTAB_activated	33	TC_TX_packets	60	actionType	87	OVS_mean_total
7	IPTAB_complexity	34	encapsulationLayer	61	actionName	88	OVS_median_total
8	IPTAB_maxRules	35	encapsulationType1	62	IPTAB_min_crr_currentMatchedPkts	89	OVS_st_total
9	IPTAB_rx_bytes	36	encapsulationType2	63	IPTAB_max_crr_currentMatchedPkts	90	OVS_q1_total
10	IPTAB_rx_packets	37	sense	64	IPTAB_min_total_totalMatchedPkts	91	OVS_q3_total
11	IPTAB_tx_bytes	38	l3Protocol	65	IPTAB_max_total_totalMatchedPkts	92	TC_min_crr_currentMatchedPkts
12	IPTAB_tx_packets	39	dstIP	66	IPTAB_numberRulesActivatedTotal	93	TC_max_crr_currentMatchedPkts
13	OVS_activated	40	macSrc	67	IPTAB_mean_crr	94	TC_min_total_totalMatchedPkts
14	OVS_complexity	41	macDst	68	IPTAB_median_crr	95	TC_max_total_totalMatchedPkts
15	OVS_maxRules	42	l4Protocol	69	IPTAB_st_crr	96	TC_numberRulesActivatedTotal
16	OVS_rx_bytes	43	tos	70	IPTAB_q1_crr	97	TC_mean_crr
17	OVS_rx_dropped	44	outTos	71	IPTAB_q3_crr	98	TC_median_crr
18	OVS_rx_packets	45	dstPort	72	IPTAB_mean_total	99	TC_st_crr
19	OVS_tx_bytes	46	state	73	IPTAB_median_total	100	TC_q1_crr
20	OVS_tx_dropped	47	totalpktCount	74	IPTAB_st_total	101	TC_q3_crr
21	OVS_tx_packets	48	totalBits	75	IPTAB_q1_total	102	TC_mean_total
22	TC_activated	49	packetSize	76	IPTAB_q3_total	103	TC_median_total
23	TC_queueDiscipline	50	programmableTechnology	77	OVS_min_crr_currentMatchedPkts	104	TC_st_total
24	TC_queueLenght	51	activatedRuleTimeSecs	78	OVS_max_crr_currentMatchedPkts	105	TC_q1_total
25	TC_complexity	52	averageMatchedBytes	79	OVS_min_total_totalMatchedPkts	106	TC_q3_total
26	TC_crr_bwd_guaranteed	53	averageMatchedPackets	80	OVS_max_total_totalMatchedPkts	107	timestamp
27	TC_maxRules	54	currentMatchedBytes	81	OVS_numberRulesActivatedTotal		

**Table 3:** Description of boolean features in IW-IB-5GNET dataset.

Feature Name	Representation	Description
IPTAB_activated	[True, False]	Presence of iptables in the interface.
OVS_activated	[True, False]	Presence of ovs in the interface.
TC_activated	[True, False]	Presence of linux tc in the interface.
Sense	[ingress, egress]	Direction of network traffic flow.

**Table 4:** Description of metadata features in W-IB-5GNET dataset.

Feature Name	Data Type	Categorical Values	Additional Info
Hostname	text	-	Data extraction host name.
AbstractionLayer	number	[0, 1, 2]	Level of virtualization.
Iface	text	-	Interface name where data extraction was performed.
Iface_speed	number	-	Network interface speed.
IPTAB_complexity	number	[8]	Level of complexity iptables rules.
IPTAB_maxRules	number	[4096]	iptables rule limit.
OVS_complexity	number	[4]	Level of complexity ovs rules.
OVS_maxRules	number	[16,384]	ovs rule limit.
TC_queueDiscipline	text	[noqueue, fq_codel, atm, htb, prio]	Primary iface qdisc queue discipline.
TC_queueLenght	number	-	Max number of packets allowed in the queue.
TC_complexity	number	[8]	Level of complexity tc rules.
TC_maxRules	number	[4096]	tc rule limit.
encapsulationLayer	number	[0, 1, 2]	Number of encapsulations of a traffic flow.
encapsulationType1	number	[gtp, vxlan]	Type of first encapsulation.
encapsulationType2	number	[gtp, vxlan]	Type of second encapsulation.
L3Protocol	number	[ipv4, ipv6, icmp, arp]	Layer 3 protocol of flow.
dstIP	number	-	Flow destination IP.
macSrc	number	-	Flow source mac address.
macDst	number	-	Flow destination mac address.
L4Protocol	number	[tcp, udp]	Layer 4 protocol of flow.
Tos	number	[0]	Type of service.
OutTos	number	[0]	Out type of service.
dstPort	number	-	Flow destination port.
State	text	[active, dropped, inactive]	Flow state description.
programmableTechnology	text	[TC, OVS, IPTABLES]	Data-plane technology used to do the action.
ruleComplexity	number	[1, 2, 3…]	Rule performance complexity.
actionType	text	[INSERT, SET, DELETE]	Definition of action type.
actionName	text	[DROP, PRIORITY, QUEUE, SLICE]	Definition of action name.

**Table 5:** Use-case examples in which to use the IW-IB-5GNET dataset.

	Purpose	Use-Case Description	Target	Relevant Features
Management	Security	Anomaly detection. Monitoring of unusual activities to respond to security incidents.	0: Benign traffic 1: Malicious traffic	34 to 49
	Traffic	Balancing traffic load across different network interfaces to prevent congestion.	Interface	4 to 49
	QoS	Defining QoS policies according to the status of the network and active network policies based on user intents.	QoS policy definition	6 to 33 62 to 106
Optimisation	Resource	Detecting redundant, unused rules to reduce processing overhead and policy congestion.	0: keep active rule 1: delete/change active rule	50 to 106
	QoS	Predictive modeling to determine the best technology/method for implementing and enforcing active network policies.	Optimal technology for active policy	34 to 61

**Table 6:** Statistical values of boolean features in IW-IB-5GNET dataset.

Feature Name	Count	Unique	Top	Freq
IPTAB_activated	64,290	2	False	55,569
OVS_activated	64,290	1	True	64,290
TC_activated	64,290	1	True	64,290
sense	64,290	2	ingress	55,569

**Table 7:** Descriptive statistics values of numerical features in IW-IB-5GNET dataset.

Feature	Unit	Mean	Std	Min	Max
ContextSwitchesPerSecond	Switch/s	3.78	2.65	0	9
IPTAB_RX_bytes	Bytes/s	178,080	495,268	0	3,524,780
IPTAB_RX_packets	Packets/s	987	2661	0	14,866
IPTAB_TX_bytes	Bytes/s	4,031,111	13,392,294	0	184,598,347
IPTAB_TX_packets	Packets/s	5345	17,555	0	151,650
OVS_RX_bytes	Bytes/s	9,290,003	12,603,834	9926	78,227,821
OVS_RX_packets	Packets/s	20,772	18,092	93	72,736
OVS_TX_bytes	Bytes/s	2,135,533	9,334,686	6354	156,582,130
OVS_TX_packets	Packets/s	5014	16,145	611	145,377
TC_RX_bytes	Bytes/s	2,642,547	7,151,074	0	62,570,352
TC_RX_dropped	Packets/s	5145	9556	0	57,989
TC_RX_packets	Packets/s	5682	10,507	0	58,842
totalpktCount	Packets	6866	3809	840	18,120
totalBits	Bits	21,578,431	25,187,493	416,976	148,316,160
packetSize	Bytes	395	355	32	1024
activatedRuleTimeSecs	Seconds	59	36	1	144
averageMatchedBytes	Bytes/s	40,171	37,985	3665	785,862
averageMatchedPackets	Packets/s	89	39	42	969
currentMatchedBytes	Bytes/s	37,469	91,559	0	9,513,560
currentMatchedPackets	Packets/s	83	159	0	11,859
totalMatchedBytes	Bytes	2,299,883	2,792,487	5772	16,381,370
totalMatchedPackets	Packets	5031	3518	70	14,855

## Data Availability

Data available upon request due to privacy restrictions.

## Supplementary Materials

Excerpt from the IW-IB-5GNET dataset: https://github.com/jimenaandrade/iw-ib-5gnet (accessed on 28 November 2023).

## Abbreviations

The following abbreviations are used in this manuscript:

AI	Artificial Intelligence
AN	Autonomous Network
B5G	5G-and-beyond network
ML	Machine Learning
SDN	Software-Defined Networking
IBN	Intent-Based Network
SLA	Service-level Agreement
IDS	Intrusion Detection System
DoS	Denial of Service
DDoS	Distributed Denial of Service
RAN	Radio Access Network
ISP	Infrastructure Service Provider
DSP	Digital Service Provider
RIA	Resource Inventory Agent
SMA	Security Monitoring Agent
RMA	Resource Monitoring Agent
CPM	Cognitive Policy Manager
FCA	Flow Control Agent
CORE	Common Open Research Emulator
UDP	User Datagram Protocol
